# Clinical and Functional Outcomes of Kinematic Aligned Total Knee Arthroplasty with a Medial Pivot Design: Two-Year Follow-Up

**DOI:** 10.3390/jcm12237258

**Published:** 2023-11-23

**Authors:** Corrado Sosio, Nicolò Rossi, Paolo Sirtori, Ricardo Ciliberto, Michele Davide Maria Lombardo, Giuseppe Michele Peretti, Laura Mangiavini

**Affiliations:** 1 IRCCS Istituto Ortopedico Galeazzi, 20161 Milan, Italy; 2 Residency Program in Orthopaedics and Traumatology, University of Milan, 00133 Milan, Italy; 3 Department of Biomedical Sciences for Health, University of Milan, 00133 Milan, Italy

**Keywords:** total knee arthroplasty, kinematic alignment, medial pivot, knee osteoarthritis

## Abstract

Background: Kinematic alignment (KA) restores native limb alignment following total knee arthroplasty (TKA). The association of this technique with a medial pivot implant design attempts to re-establish the physiological kinematics of the knee. This study aims to analyze the clinical and radiological outcomes of patients undergoing MP-TKA with kinematic alignment and to assess the effect of limb alignment on the clinical outcomes. Methods: We retrospectively analyzed 55 patients who underwent kinematic aligned medial pivot TKA from September 2018 to January 2020. Patient-related outcomes (PROMs) were collected at baseline, 3, 12, and 24 months after surgery. Long-standing weight-bearing radiographs were performed three months after surgery. Results: We demonstrated a significant improvement in clinical outcomes from 3 months after surgery up to 24 months of follow-up. This clinical improvement was independent of limb alignment. The radiological analysis showed that the patient’s native limb alignment was restored and that their joint line orientation was parallel to the floor. Conclusion: The association of kinematic alignment and a medial pivot TKA implant allows for a fast recovery, with good clinical and functional outcomes up to a minimum of 2 years of follow-up, independent of the final limb alignment.

## 1. Introduction

Knee osteoarthritis (KOA) is a common musculoskeletal disorder causing pain and dysfunction of the lower limbs [1]. This chronic condition leads to progressive damage of the articular cartilage. Nonetheless, OA should not be considered just as a “cartilage disease” but a disease affecting the entire environment of the joint space. In more advanced stages of the disease, different elements, such as the synovial membrane and the subchondral bone, play a pivotal role in OA pathogenesis. OA is a disabling condition that considerably impacts patients’ quality of life, leading to a severe socioeconomic burden [2]. It has been estimated that the prevalence of hip and knee OA is around 300 million cases worldwide, and in 2010, the Global Burden of Disease study found hip and knee OA to be one of the leading causes of global disability [3,4]. Different non-surgical treatment options are available for mild–moderate KOA [5]. Total knee arthroplasty (TKA) is considered as a first choice of treatment (Kellgren–Lawrence ≥ 3) to alleviate pain and restore the quality of life in patients with severe joint disease [6]. Different studies have highlighted that the long-term survival rate of TKA can reach more than 90% after 15 years [7,8]. However, the number of unsatisfied patients is still high, with a rate between 20 and 25% [9]. Mechanical alignment (MA) technique has extensively been used for performing TKA due to successful clinical and radiological outcomes. The primary goal of MA TKA was the achievement of a stable knee with a neutrally aligned lower limb. Therefore, the historical aim of the MA technique is to reproduce a biomechanically friendly prosthetic knee without taking into consideration constitutional patient-specific alignment [10]. Conversely, kinematic alignment (KA) is a novel philosophy applied to total knee arthroplasty (TKA) that aims to co-align the prosthetic components with the three kinematic axes of the knee, which are the transverse axis of the femur, the transverse patellar axis, and the longitudinal axis of the tibia [11,12]. This patient-specific approach strives to reproduce the natural kinematic of each specific knee to overcome the unacceptable number of unsatisfied patients who do not perceive an enhancement in their quality of life following the MA TKA technique [9,13]. This surgical technique, described by Howell, is a personalized joint line reconstruction of the articular surface through calipered verified bone cuts, without ligament release [14,15]. This technique re-establishes the constitutional (native) alignment of the limb by restoring the original bone morphotype of the knee and maintaining the natural tension of the soft tissues. In recent years, several studies have demonstrated comparable or better early- and mid-term clinical results in patients undergoing TKA with kinematic alignment compared to mechanical alignment [16,17,18,19,20,21,22,23]. Moreover, Howell et al. showed that limb alignment outside of the neutral range, or the implantation of the tibial component in a varus position, did not affect implant survival at longer periods of follow-up, with good clinical outcomes [24]. 

However, normal knee kinematics is complex as it requires the integrity of the cruciate ligaments, which antero-posteriorly stabilize the knee and guide its rotation. Knee motion occurs both on the transverse axis and longitudinal axis, the latter driving the rotation of the tibia on the femur. The study by Freeman and Pinskerova showed that the medial femoral condyle behaves as a sphere, which rotates but does not translate on a concave tibial plate, whereas, the lateral condyle behaves like a wheel on ice, rolling but also antero-posteriorly sliding on a flat tibial plate [25]. This asymmetric movement during flexion–extension causes a rotation of the knee around a longitudinal axis passing through the center of the medial sphere. In a retrospective comparison, Samy et al. showed better results in total knee implants reproducing the medial pivot pattern (MP-TKA) compared to posteriorly stabilized (PS) implants in terms of deep knee flexion and stability. The authors stated that the MP-TKA design improves anteroposterior stability due to the elevated medial anterior and posterior lips of the polyethylene liner. Moreover, this implant allows for a good rotation and flexion of the knee due to the lack of lateral constraints [25]. In two groups of patients implanted with the kinematic alignment technique, Scott et al. also demonstrated the superiority of a medial-stabilized device over a posterior-stabilized device in terms of mid-flexion stability [26].

In this study, we retrospectively analyzed patients undergoing TKA, combining a kinematic alignment technique with an implant specifically designed to reproduce the physiological medial pivoting pattern. The association of a surgical procedure that restores the bone morphotype and natural ligament laxities of the knee with a prosthesis that allows for antero-posterior stability and proper axial rotation should reproduce a more physiological kinematic of the knee, possibly resulting in better functional outcomes.

The primary objective of the study was to evaluate the clinical and functional outcomes for a consecutive series of patients treated with a kinematic aligned medial pivot TKA, measured at up to two years of follow-up. The secondary objective was to test the effect of limb alignment on the clinical results.

## 2. Materials and Methods

### 2.1. Study Design

We performed a clinical and radiological retrospective study of 55 patients treated with a kinematic aligned medial pivot TKA and enrolled in our internal prosthesis registry (Datareg) from September 2018 to January 2020.

Inclusion criteria were:

- Patients with disabling knee pain and functional loss with evidence of Kellgren–Lawrence grade III or IV knee osteoarthritis;
- Any severity of varus or valgus and flexion preoperative deformities;
- Any grade of varus or valgus postoperative deformity;
- Availability of preoperative and postoperative X-rays (antero-posterior, latero-lateral, Merchant, long-standing weight-bearing views);
- Availability of preoperative and postoperative (3, 12, and 24 months) patient-related outcome measures (PROMs):
  1. Visual Analogue Scale (VAS);
  2. 12-Item Short Form Survey Physical Scale (SF-12-PS);
  3. 12-Item Short Form Survey Mental Scale (SF-12-MS);
  4. Knee Injury and Osteoarthritis Outcome Score—Physical Function Short Form (KOOS-PS);
  5. Knee Society Score—Clinical Score (KSS-CS);
  6. Knee Society Score—Functional Score (KSS-FS);
  7. Forgotten Joint Score (FJS).

Exclusion criteria were:

- Previous surgery or traumatic fracture on the lower limb that altered the constitutional mechanical axis;
- An ankle or hip replacement on the treated limb;
- Collateral ligament laxity.

### 2.2. Surgical Technique

All patients were implanted with a cemented medial pivot TKA (MP-TKA: GMK Sphere—Medacta) with the excision of the posterior cruciate ligament (PCL). This implant accurately reproduces the medial compartment with a ball-in-socket design and the lateral compartment due to a flat insert. All implantations were performed using the calipered verified kinematic alignment technique with a conventional instrument proposed by Howell et al. [12]. The surgical sequences consisted of three steps: (a) a distal femoral cut that matches the distal thickness of the implant, after correction for cartilage wear and kerf of the blade, (b) a tibial proximal cut that is fine-tuned in distalization and varus–valgus using bone recuts to obtain an adequate and symmetric extension space, and (c) a posterior femoral cut that matches the posterior thickness of the implant after correction for cartilage wear and the kerf of the blade. At each step, the correct amount of bone and cartilage removed was checked using a caliper and eventually adjusted. Before the posterior femoral cut, the flexion and extension gaps were matched: in the case of a tight gap in flexion, the tibial slope was increased, while in case of a loose gap in flexion, the tibial slope was reduced. Furthermore, in selected cases characterized by too loose of a gap in flexion, the “virtuous mistake” technique described by Malavolta et al. was adopted [27]. Thus, the balance and matching of the gaps were obtained only with bone recuts without ligament release. The patella was never resurfaced but only treated with a plasty.

### 2.3. Radiological Analysis

Limb alignment was measured on long-standing weight-bearing radiographs performed in a “stand at attention” modality. All measurements were calculated by two independent examiners and mean values were used for the evaluation. 

The following angles were evaluated:

- HKA (hip—knee—ankle) angle;
- mLDFA (mechanical lateral distal femoral angle);
- MPTA (medial proximal tibial angle);
- JLOA (joint line orientation angle).

**Figure 1:**
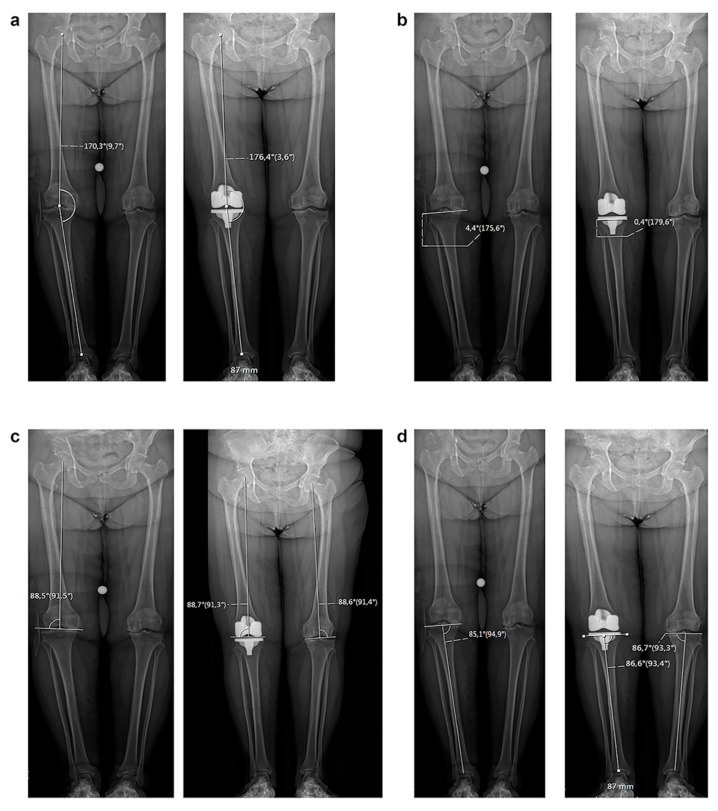
Radiological evaluation of HKA (**a**), JLOA (**b**), mLDFA (**c**), and MPTA (**d**) on the treated limb. mLDFA (**c**) and MPTA (**d**) were also measured on the contralateral limb.

The mechanical lateral distal femoral angle and medial proximal tibial angle were also measured on the contralateral limb with the exception of patients with previous hip, knee, or ankle replacements or fractures that altered the mechanical angles (Figure 1).

### 2.4. Clinical and Functional Analysis

Patients were systematically evaluated at baseline, 3, 12, and 24 months after surgery with specific PROMs, and the results were reported in the internal prosthesis register. VAS, SF12-PS, SF12-MS, KOOS-PS, KSS-CS, and KSS-FS scores were collected at each time point. FJS was only evaluated at the postoperative time points.

### 2.5. Statistical Analysis

The analyses were performed using R software v4.0.3 (R Core Team, Wien, Austria). Data distributions were assessed using the Shapiro–Wilk normality test. According to the results of this test, one-way ANOVA or a Kruskal–Wallis test (or repeated measures ANOVA/Friedman test in the case of paired data) with the appropriate post-tests were used to assess the differences among more than two groups (or time points). Similarly, Student’s *t*-test or Mann–Whitney test (or a paired *t*-test and Wilcoxon test for paired data) were used to assess differences between two groups. A two-way ANOVA test was used to measure the possible interaction between two variables (group and time point). Multiple linear models were developed to assess the influence of demographic and radiological data on patient-reported outcome measures (PROMs). Gvlma function in R was used to assess the value of the linear models’ assumptions in each model. *p* values < 0.05 were considered statistically significant. 

## 3. Results

All patients undergoing a kinematic aligned medial pivot TKA from September 2018 to January 2020 were enrolled, and thus 55 patients were analyzed in this study. Preoperative patient demographics are reported in Table 1. Furthermore, we did not observe any septic or aseptic loosening complications at the end of follow-up; none of the patients required a second operation.

### 3.1. Patient-Reported Outcome Measures (PROMs)

A significant improvement in PROMs was recorded at all follow-up times compared to baseline values. PROMs values at baseline, 3, 12, and 24 months after surgery are reported in Table 2. VAS evaluation showed a statistically significant decrease between baseline and 3 months of follow-up (7.5 vs. 3, respectively). Conversely, this value remained stable at 1 year and 2 years of follow-up. As far as the other PROMs are concerned, no significant differences were found between 3-, 12-, and 24-month follow-ups with the exception of KSS-CS (Table 2). KSS-CS showed a mean value of 42 at baseline and an increase up to 94.0 at 24 months. FJS scores significantly increased between 3, 12, and 24 months after surgery, with a value of 79.17 at 3 months and 89.6 at 2 years of follow-up. PROMs were not significantly influenced by postoperative alignment (varus, neutral, valgus) nor by tibial component orientation (varus or not varus), independent of BMI, age, or sex.

### 3.2. Radiological Evaluation

**Figure 2:**
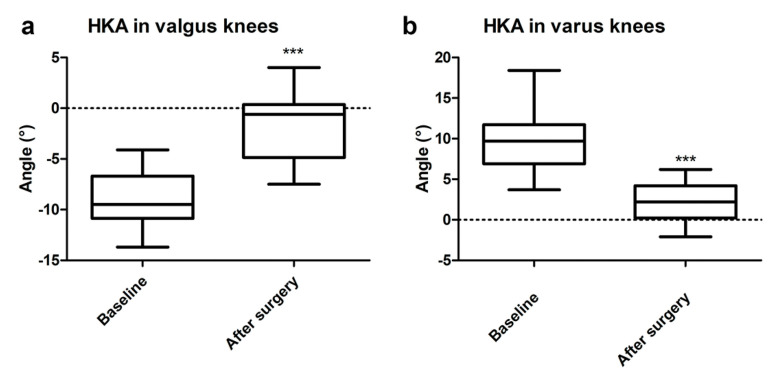
HKA comparison in valgus (**a**) and varus (**b**) knees at baseline and after surgery. Values are expressed by median and confidence intervals: *** *p* < 0.001.

A significant difference between the preoperative and postoperative HKA angles was registered, both in valgus and varus knees (*p* < 0.001, *p* < 0.001, respectively) (Figure 2). The postoperative alignment of the limb was neutral (0 ± 3°) in 58% of the patients, varus (>3°; maximum = 6.2°) in 33% of the patients, and valgus (>3°; maximum = 7.5°) in 9% of the patients.

**Figure 3:**
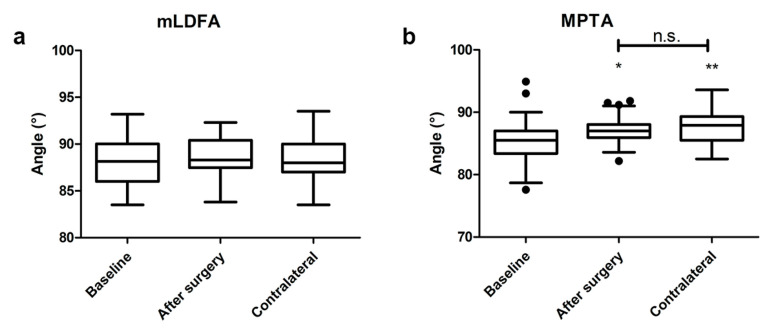
mLDFA (**a**) and MPTA (**b**) comparison between baseline, after surgery, and with the contralateral limb. Values are expressed by median and confidence intervals: * *p* < 0.05, ** *p* < 0.01, n.s.—not statistically significant.

No significant difference was found between preoperative and postoperative mLDFA, as well as between the mLDFA in the operated limb compared to the contralateral mLDFA (overall *p* = 0.589) (Figure 3). The MPTA was significantly increased after surgery (*p* < 0.05); however, the MPTA in the operated limb was comparable with the contralateral MPTA (*p* = 0.430) (Figure 3).

**Figure 4:**
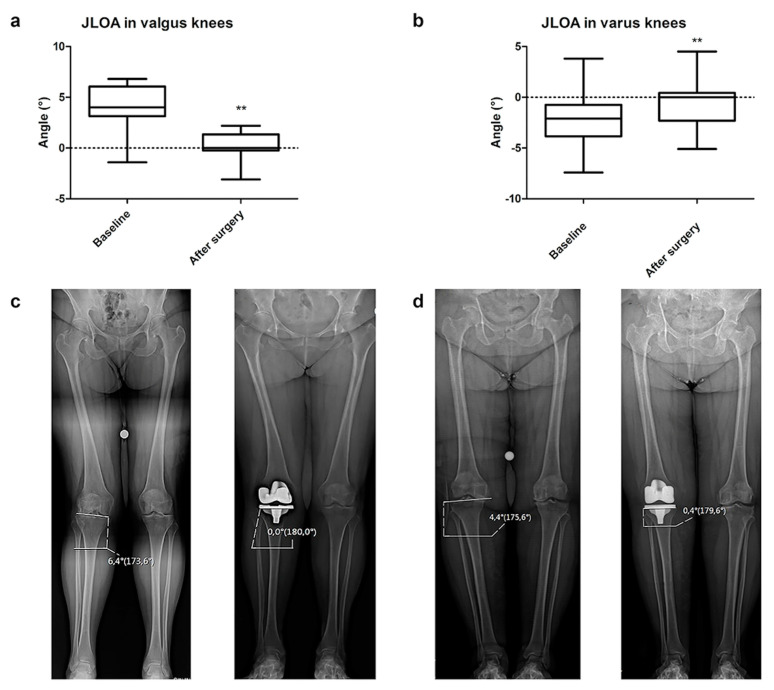
JLOA comparison in valgus (**a**) and varus (**b**) knees at baseline and after surgery. Radiological examples of JLOA measurements in valgus (**c**) and varus (**d**) knees are shown in (**c**,**d**). Values are expressed by median and confidence intervals: ** *p* < 0.01.

Furthermore, a significant difference between the preoperative and postoperative obliquity of the joint line (JLOA) was detected, in both valgus and varus deformity (*p* < 0.001, *p* < 0.001, respectively). The postoperative JLOA values got close to 0° both in valgus and varus final alignments (Figure 4).

## 4. Discussion

In recent decades, researchers have focused their attention on reproducing the natural kinematics of each specific knee with the aim of reducing the rate of unsuccessful TKA. The KA technique has been recently introduced to align the prosthetic components with the three kinematic axes of the knee, aiming to create a more patient-specific approach [11]. However, the debate on the best alignment option is still open, and a solution has yet to be found. Our study demonstrated that patients who underwent a kinematic-aligned medial pivot TKA achieved good clinical and functional results 3 months after surgery, suggesting a fast postoperative recovery. This could be due to a procedure that guides bone removal based on implant thickness and preserves soft tissues. The calipered verified technique proposed by Howell has demonstrated lower surgical invasiveness than mechanical alignment [28]. Our results at three months support the evidence that this technique reduces postoperative pain and swelling, thus optimizing joint function and accelerating recovery [29,30]. These data are in line with the most recent studies in the literature. A recent review by Gao et al. demonstrated that the incidence of ligament release in the MA-TKA group was higher than that in the KA-TKA group and that the KA-TKA group had better clinical outcomes and knee range of flexion than the MA-TKA group at short-term follow-up [31]. Moreover, most of these results remained stable up to 24 months of follow-up, confirming the hypothesis that kinematic aligned medial pivot TKA leads to stable outcomes.

Interestingly, our study showed that the FJS has high values at three months that significantly increase up to 24 months. To better describe the patients’ perception after TKA implantation, a variety of patient-reported outcome measures (PROMs) have been introduced and are currently used in the clinical setting. These PROMs evaluate clinical and functional outcomes other than patient satisfaction and quality of life restoration [32]. In addition, FJS is a score that assesses a patient’s ability to forget about their artificial joint in everyday life, showing a progressive achievement of the ideal objective of a forgotten joint [33]. The feeling of a more natural knee may be explained by the combination of a kinematic alignment concept and a prosthesis with medial pivot. Calipered KA set the femoral and tibial components to restore the patient’s prearthritic joint lines without the release of ligaments; resurfacing the knee closely coaligns the prosthetic components with the three kinematic axes of the native knee, reducing the risk of kinematic conflict between the components and soft tissues. The medial pivot design was developed to better mimic the natural kinematic of the knee, specifically, more natural femoral rollback.

The second finding of our study is that limb alignment did not affect clinical and functional outcomes. The kinematic alignment restores the native joint line height and orientation without aiming at the neutrality of the limb alignment. Howell et al. showed that the residual varus or valgus deformity in overall limb alignment and the varus orientation of the tibial component did not negatively affect the function and survival of the prosthesis up to 16 years [24]. Thus, implant failure and the risk of a second surgery from malalignment do not represent real concerns in applying this technique. In a case-match study conducted from 2006 to 2015, Nedopil showed an early tibial component failure following patients treated with KA-TKA for an incidence of 0.3%. In this study, the tibial plate failure was caused by posterior subsidence and not varus subsidence and in those patients the setting of the slope of the tibial component was more posterior than native [34]. The posterior slope greater than the native slope slackened the flexion space that promotes the excessive anterior tibial component translation and excessive posterior rollback of the femoral component on the tibial component that causes posterior overload. In this study, we did not find any early tibial component failure. Due to the excision of the PCL that increases the flexion gap, the tibial slope was reduced in many cases, with a mean reduction of 4°. Our cohort of patients showed an increase in the native slope that reduced the risk of tibial failure. Our data showed a significant reduction in preoperative limb deformity, both in valgus and varus positions, but the postoperative alignment of the limb was only neutral (0 ± 3°) in 58% of the patients, with a high number of outlier alignments. However, we achieved an accurate reproduction of native mechanical angles confirming the symmetry between the right and left mechanical angles on the frontal plane that occurred in 97% of the patients [35].

Moreover, mLDFA was not significantly different from the preoperative angles, confirming the accuracy of the calipered technique in reproducing the knee morphotype without any intraoperative radiological reference, navigation system or robotic technique. Surprisingly, the postoperative MPTA was significantly different compared to the preoperative values. Seventy percent of our patients displayed a preoperative varus knee alignment, and a subgroup of these patients also had a high degree of metaphyseal deformity, with a value of MPTA below 87°. In this subgroup, the metaphyseal deformity is usually caused by bone wear, following the study of [36]. In our technique, a native MPTA is restored by compensating the cartilage and bone wear. Indeed, the tibial cut is parallel to the femoral cut using the natural tension of the collateral ligaments. The comparison with the contralateral limb confirmed the re-establishment of a native MPTA that did not show any statistical difference in MPTA values, confirming the symmetry on the frontal plane that occurred in 97% of patients [35]. 

**Figure 5:**
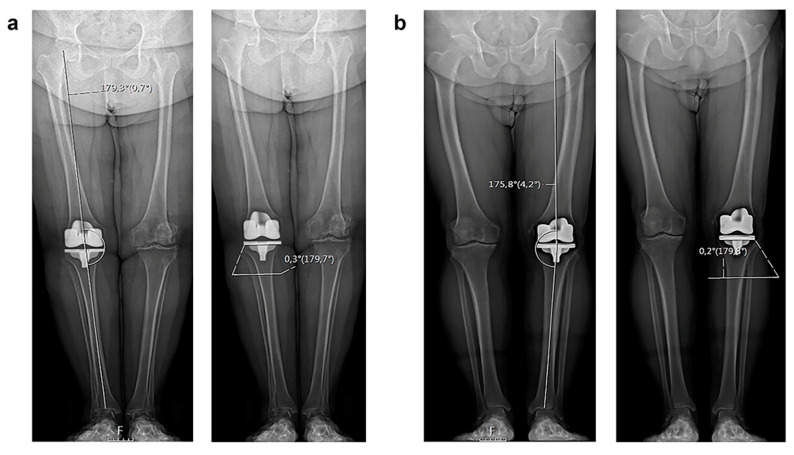
Radiological examples of the parallelism of the joint line both in neutral (**a**) and varus (**b**) final limb alignment.

Finally, we also analyzed the orientation of the postoperative joint line on long-standing weight-bearing radiographs. In patients with preoperative varus and valgus deformities, the knee joint line was inclined laterally or medially, respectively. After surgery, the orientation of the joint line was parallel to the floor when standing, independent from the preoperative limb alignment [37,38] (Figure 5). Victor et al. demonstrated that in non-arthritic patients, the joint line was relatively parallel to the floor both in patients with neutral and constitutional varus alignment. This orientation allows for an ideal joint loading distribution and for a reduction in shear stresses [39]. Thus, restoring the joint line parallel to the floor may also be beneficial in a prosthetic knee, reducing the rate of implant wear or mobilization. 

This study has some limitations. First, in our retrospective analysis, the control group was missing. Thus, we cannot compare the clinical and functional outcomes of KA with the results of other techniques. Prospective studies with a comparison group are therefore advisable to definitively prove the efficacy of the KA. In addition, our study analyzed only three time points up to a period of two years. Nevertheless, our results are comparable to the recent literature on kinematic alignment [15]. Lastly, additional analyses with longer follow-ups are required to confirm these data and evaluate the survivorship of the implant.

## 5. Conclusions

This study suggests that the association of the kinematic alignment technique with a medial pivot TKA implant in the treatment of knee osteoarthritis allows for a fast recovery and good clinical and functional outcomes, which are kept stable at a minimum two years of follow-up, independent from postoperative limb alignment. The retrospective nature of the study combined with the absence of a comparison group does not allow for any solid conclusions to be drawn, but it reflects the findings in the most recent literature. Further studies with a longer follow-up and a direct comparison between the two surgical approaches are required. 

## Figures and Tables

**Table 1:** Demographic characteristics of the patients.

Age (years)	71.5 (±8.4)
BMI (kg/m^2^)	29.3 (±4.6)
Sex	32 F, 23 M
Side	32 R, 23 L

**Table 2:** PROMs values at baseline, 3, 12 and 24 months after surgery. Values are expressed by median and interquartile range. * *p* < 0.05, ** *p* < 0.01, *** *p* < 0.001 vs. baseline ^###^ *p* < 0.001 vs. 3 months, ^@^ *p* < 0.001 vs. 3 months, ^§^ *p* < 0.001 vs. 12 months, n.a.—not applicable.

	Baseline	3 Months	12 Months	24 Months
VAS	7.5 (6.0–8.25)	3.0 (1.0–5.0) ***	2.0 (0.0–5.0) ***	2.0 (0.0–5.0) ***
SF12-PS	32.2 (27.4–41.6)	44.4 (37.6–52.1) ***	46.7 (37.1–53.6) ***	46.8 (37.2–53.8) ***
SF12-MS	48.1 (39.7–56.8)	57.2 (48.2–60.8) **	51.0 (45.1–60.7) *	51.0 (45.1–60.7) *
KOOS-PS	48.5 (42.0–62.0)	33.6 (27.5–39.5) ***	31.8 (16.7–38.6) ***	33.6 (27.5–39.5) ***
KSS-CS	42.0 (29.0–52.0)	80.0 (68.0–88.5) ***	93.5 (85.0–98.0) ***^, ###^	94.0 (85.5–98.5) ***^, ###^
KSS-FS	45.0 (40.0–60.0)	90.0 (72.5–100.0) ***	100.0 (80.0–100.0) ***	91 (73.0–100.0) ***
FJS	n.a	79.17 (75.00–83.33)	83.30 (81.30–87.5) ^###^	89.6 (84.8–92.6) ^@, §^

## Data Availability

Data are deposited in a public repository (OSF) and available at this link: https://osf.io/b5hdp/?view_only=9f04dacd1fe44c5da6c8bd4f8c316109 (accessed on 18 October 2023).
